# Multistep reconstruction of a post-traumatic defect in the lower limb with AV loop and free myocutaneous latissimus dorsi flap combined with a perforator monitor skin island after loss of ALT flap

**DOI:** 10.3205/iprs000155

**Published:** 2021-06-09

**Authors:** Tobias Summer, Mazen Abou Mrad, Olimpiu Bota, Kevin Bienger, Adrian Dragu

**Affiliations:** 1 Department for Plastic and Hand Surgery, UniversitätsCentrum für Orthopädie und Unfallchirurgie, Universitätsklinikum Carl Gustav Carus an der Technischen Universität Dresden, Germany; 2 Medical University of Lodz, Poland

**Keywords:** reconstruction, lower limb, microsurgery, plastic surgery, latissimus dorsi

## Abstract

Crush injuries of the lower extremity with extensive osseous and soft tissue damage impose a big challenge even for an interdisciplinary reconstructive approach. Multistep reconstruction with negative wound pressure therapy for soft tissue management and external fixation for osseous stability preceding free flap transfer leads to optimized outcome. We report the successful multistep reconstruction of a third-degree open right tibial fracture with extensive soft tissue defect with an arteriovenous loop preceding latissimus dorsi flap coverage with a perforator skin island after loss of an anterior lateral thigh (ALT) flap due to intima damage of the recipient vessels. The described method is a safe reconstructive concept after primary flap loss with persistent extensive tissue damage.

## Zusammenfassung

Quetschverletzungen der unteren Extremität mit ausgedehnter Knochen- und Weichteilschädigung stellen selbst für interdisziplinäre rekonstruktive Konzepte eine große Herausforderung dar. Eine sequenzielle Herangehensweise, bestehend aus einem Unterdruckverband gestützten Weichteilmanagement sowie einer Knochenstabilisierung mittels Fixateur externe vor freiem Gewebetransfer, führt zu einem verbessertem Outcome. Wir berichten über die erfolgreiche mehrstufige Rekonstruktion einer rechtsseitigen, drittgradig offenen Tibiafraktur mit ausgedehntem Weichteildefekt mittels einer arteriovenösen Gefäßschleife und folgendem Latissimus dorsi Lappentransfer mit einer perforatorbasierten Hautinsel nach anterior lateral thigh (ALT) Lappenverlust durch Intimaschaden der Anschlussgefäße. Die beschriebene Methode stellt ein sicheres rekonstruktives Konzept nach Lappenverlust mit konsekutiv persistierendem Gewebedefekt dar.

## Introduction

Severe injuries of the lower extremity are a life-changing event for the patient and a technical challenge for the medical team. 

Even if limb salvage is successful, the functional outcome of injuries with extensive soft tissue damage combined with massive muscle, nerval, vascular and bone injury followed by chronic infection may be unsatisfactory [1]. The leading cause for injury of the lower extremity is motor vehicle accident (34%) with crush injuries being mostly prevalently associated with open lower extremity fractures (40%). The most common long bone fracture is the tibia and fibula at 11% [2].

Crush injuries are the result of a body part being forcefully compressed between two hard surfaces whereupon compression of the muscle mass blocks the flow of blood and oxygen to tissues resulting in ischemia. Following ischemia, necrosis occurs within a few hours [3]. 

Extensive soft tissue injury combined with open fractures is associated with higher infection rates and limited osteosynthesis options. This effect is amplified without an interdisciplinary approach involving plastic surgical soft tissue management and soft tissue expertise [4]. Prompt involvement of the department of plastic surgery ensures the early development of a reconstructive plan and enhanced soft tissue management.

The interdisciplinary surgical approach has to take into consideration both the osseous damage and the limiting massive soft tissue injuries while creating a therapeutical plan to restore the unique bone architecture and static of the lower limb and to reconstruct the nerval, muscular and vascular structures achieving optimal functionality. This should be done as early as possible in order to minimize the risk of future infections and necrosis, especially when open fractures are present. When treating a multi-trauma patient suffering from a severe injury, standardized treatment protocols are based on the ATLS (advanced trauma life support) guidelines and life-threatening injuries have to be addressed first. Only after the multitrauma patient has been stabilized, a thorough orthopedic and plastic evaluation can be conducted. Whenever a patient with a severe leg injury presents in the emergency department, two principal surgical approaches should be kept in mind:

1. Multistep treatment
2. Single step and definite treatment or amputation

Treatment options should take into consideration the severity of the injury, the patient’s biological reserve, functional status preceding the injury and personal demands (if he/she is able to communicate them). For optimized decision making (salvage vs. amputation) the MESS score can be regarded as a useful tool [5].

Crush injuries are associated with a higher occurence of acute compartment syndrome. Compartment syndrome is mostly met in lower extremity injuries with more than 30% being linked to tibial fractures. Compartment syndrome is characterized by elevated pressure in an unyielding osteofascial space. Sustained elevation of tissue pressure reduces capillary perfusion below a level necessary for tissue viability, and irreversible muscle, vascular and nerve damage may occur within hours [6].

From a microsurgeon’s point of view crush injuries impose a technical challenge due to the extensive intima damage surpassing the initial injury zone. Evaluating the extent of the vascular damage is challenging; the mechanisms and possible preventions of early clotting and/or thrombosis are currently studied in several rodent models [7], [8], [9].

This report describes the surgical treatment and follow-up of a motor vehicle driver involved in a severe crush injury of the tibial part of the lower right leg with multiple fractures including a 3^rd^ degree open tibial fracture.

In such a case, it is necessary to use a free flap transfer to safely reconstruct soft tissue. Flaps are classified in many ways, the most popular of which is according to axial blood supply developed by Mathes and Nahai (axial types I–V) [10]. Among the wide range of free flaps the anterior lateral thigh (ALT) and latissimus dorsi (LD) have been the mainstay flaps in reconstructing complex defects of the lower limbs [11], [12]. 

The LD flap was used in combination with an arteriovenous (AV) loop in this case after loss of the ALT flap due to poor local blood supply and performing the microvascular anastomosis within the zone of injury. For optimized safety, the LD transfer included the creation of a single perforator-based monitor island to facilitate flap observation.

## Case description

A 30-year old male was involved in a motor vehicle accident and suffered from multitrauma including fractures of the processus spinosi of the cervical vertebra 7 and thoracic vertebrae 1 and 2, fracture of the processus transversi of the thoracic vertebrae 8 to 11, fractures of the right olecranon, the left clavicle and a third-degree open right tibial fracture with severe soft tissue defect exposing the tibial fracture zone (Schatzker C). At first entry to the combined center of traumatology and reconstructive surgery an external fixation was performed combined with a plate osteosynthesis (LCP proximal tibial plate 3,5/6, Johnson&Johnson Synthes) to ensure limb salvage. In a second procedure the osteosynthesis was planed to be replaced by a coated tibial nail (e.g. PROtect expert tibial nail, Johnson&Johnson) after the coverage of the vacuum sealed soft tissue defect and a 6-week antibiotic therapy to avoid chronic infection with subsequent osteomyelitis. Prior to the subsequently discussed operation, an attempt to cover the defect was made by performing a free ALT flap with end-to-end anastomosis to the posterior tibial artery, but ended with flap necrosis due to arterial embolism triggered by the massive intima damage in spite of temporary therapeutic anticoagulation. The decision to make an end-to-end anastomosis within the primary injury zone is debatable, even though a preoperative CT angiography showed sufficient flow of the posterior tibial artery. The clotted posterior artery was ligated after removal of the ALT flap. 

**Figure 1:**
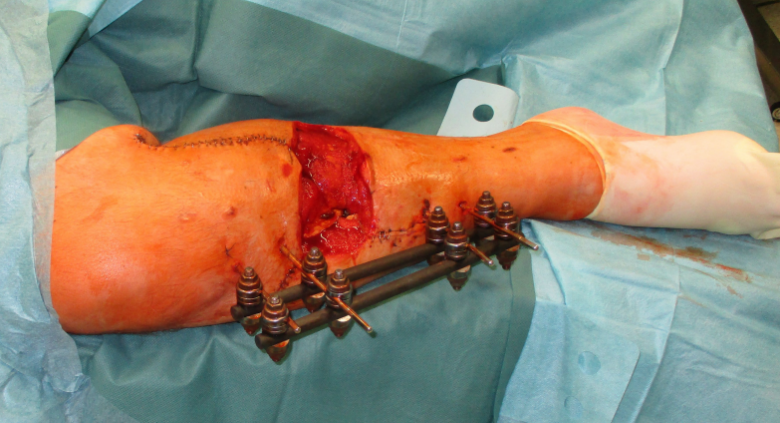
Preoperative defect

**Figure 2:**
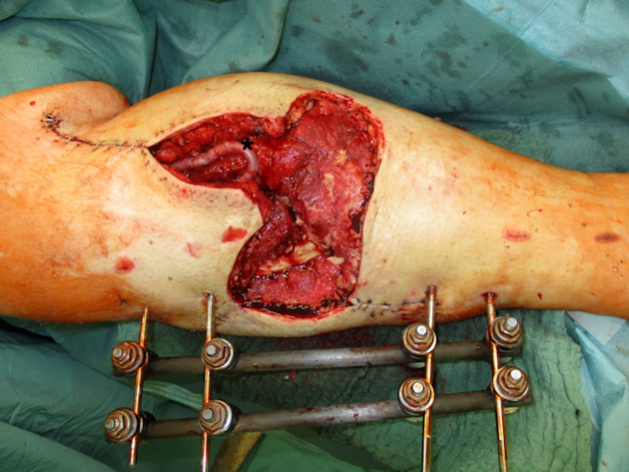
Defect with AV loop in situ

**Figure 3:**
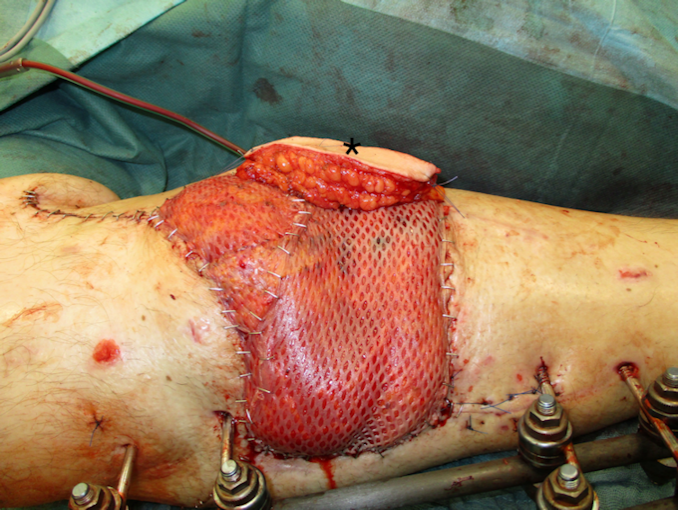
LD flap plus split skin graft and monitor island

Negative wound pressure therapy (NPWT) was used for coverage and preconditioning of the 8 x 18 cm soft tissue defect (Figure 1 (Fig. 1)). Preceding the free LD flap an AV loop system (AV loop marked * in Figure 2 (Fig. 2)) was constructed by the vascular surgery department on the popliteal P3 segment. The patient was thus ready to have the defect covered by means of a free LD flap with microvascular connection to the AV loop and simultaneous split skin coverage plus a monitor island (marked *) for better flap observation (Figure 3 (Fig. 3)) after 7 days of Doppler monitoring the AV loop. The flap showed no signs of low perfusion or infection. The monitor island was disconnected during a bedside procedure on the 7^th^ postoperative day before starting flap training via dangling of the leg. The donor sites on the left thigh and back showed no signs of seroma, wound dehiscence or inflammation.

**Figure 4:**
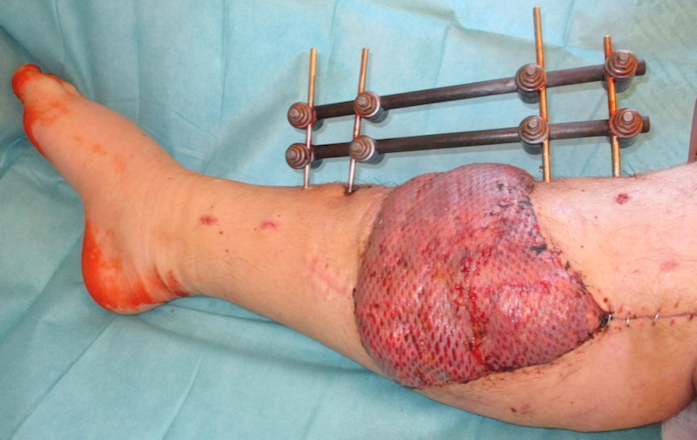
Postoperative result after 2 weeks

**Figure 5:**
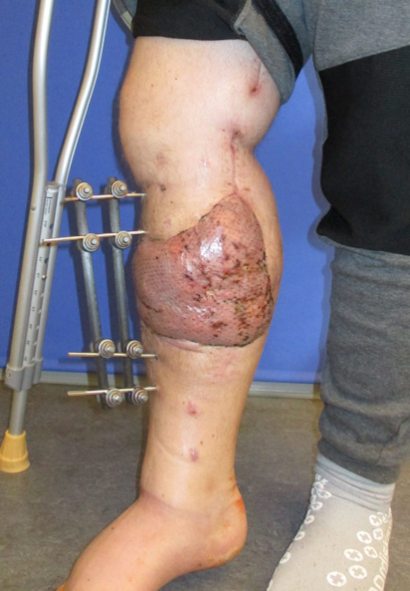
Postoperative result after 2 weeks

**Figure 6:**
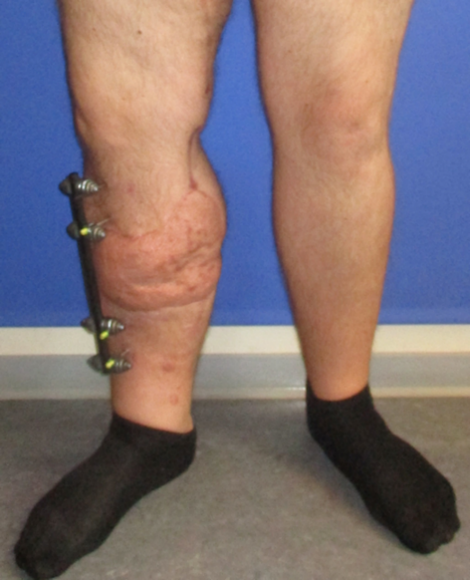
Postoperative result after 6 months, frontal view

**Figure 7:**
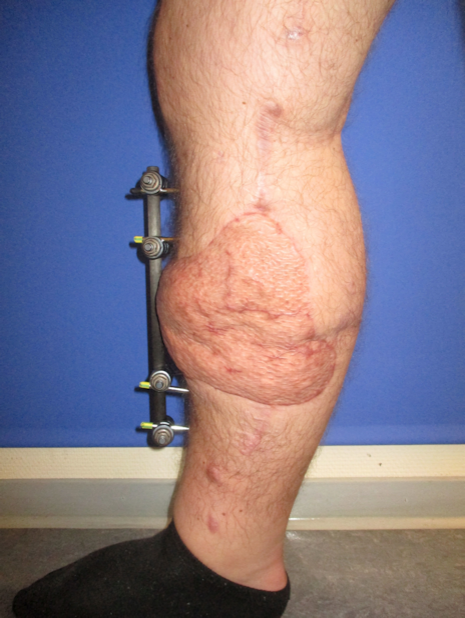
Postoperative result after 6 months, lateral view

**Figure 8:**
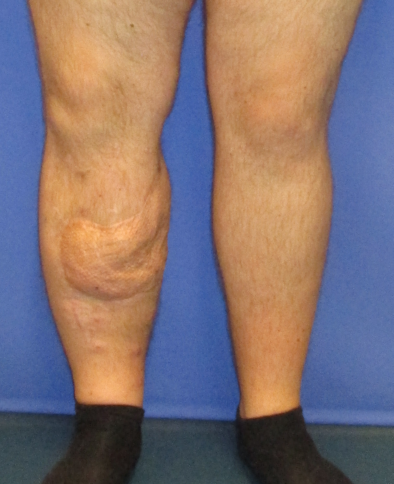
Postoperative result after 1.5 years, frontal view

**Figure 9:**
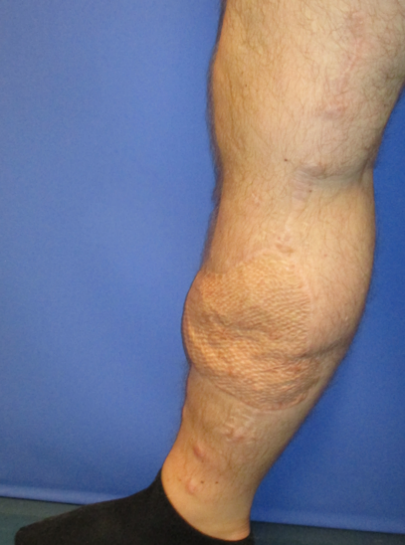
Postoperative result after 1.5 years, lateral view

**Figure 10:**
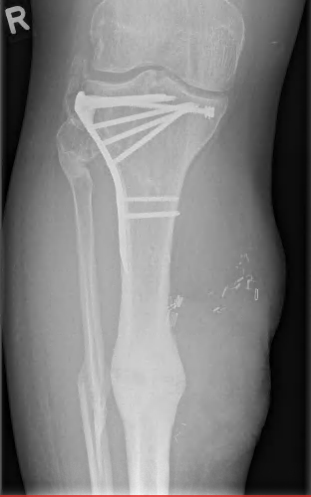
Posteropative X-rays after 1 year

The patient underwent early physiotherapy for remobilization. Customized compression garments were worn for 6 weeks for flap remodeling. He was discharged to outpatient care on the 63^rd^ day in a healthy condition with good scarring. The initial care was performed by the outpatient unit with weekly follow-up visits and short-term visits after 2 and 8 weeks (Figure 4 (Fig. 4) and Figure 5 (Fig. 5)), 6 months (Figure 6 (Fig. 6) and Figure 7 (Fig. 7)) and 1.5 years post-surgery (Figure 8 (Fig. 8) and Figure 9 (Fig. 9)). Given full weight bearing, the patient did not complain of any disturbance. A contrast CT performed at week 16 post discharge confirmed the efficacy of the AV loop (Attachment 1 ). Figure 5 (Fig. 5), Figure 6 (Fig. 6), Figure 7 (Fig. 7) and Figure 8 (Fig. 8) show the patient standing and able to walk with no crutches. The X-rays (Figure 10 (Fig. 10)) one year after bone fixation showed no sign of bone infection or pseudarthrosis after removal of the external fixator. No further surgical procedures were performed due to good overall patient satisfaction and adequate aesthetic outcome.

## Operation

**Figure 11:**
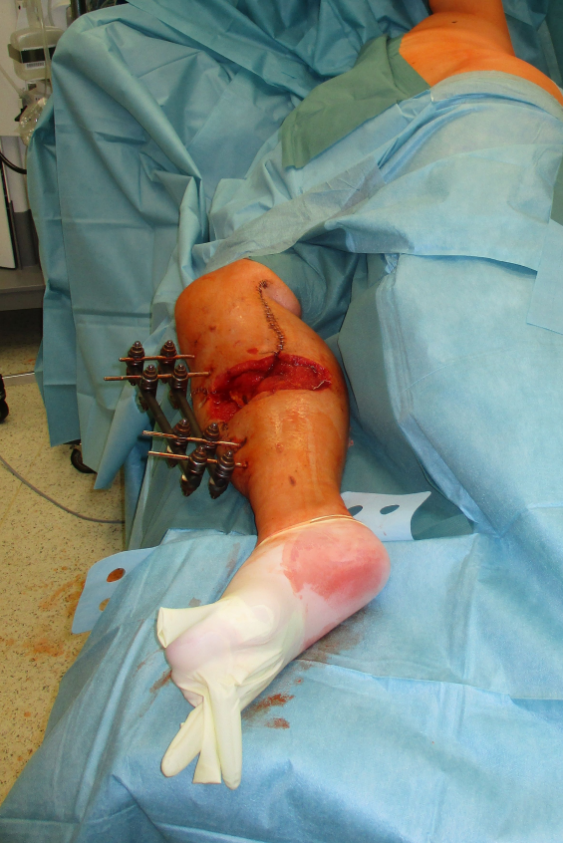
Intraoperative patient positioning

The patient was placed in a right lateral position allowing a two-team approach (Figure 11 (Fig. 11)). The AV loop (marked with *) with its arterial and venous side demonstrated a sufficient blood flow. The AV loop was placed with its vertex close to the defect with sufficient healthy tissue coverage below the knee joint thus ensuring a tension free anastomosis and preventing shearing forces which may compromise blood flow due to intima leasion. Correct positioning of the apex loop both in low mobility zones and in proximity to the defect is crucial for facilitating free flap anastomosis. 

**Figure 12:**
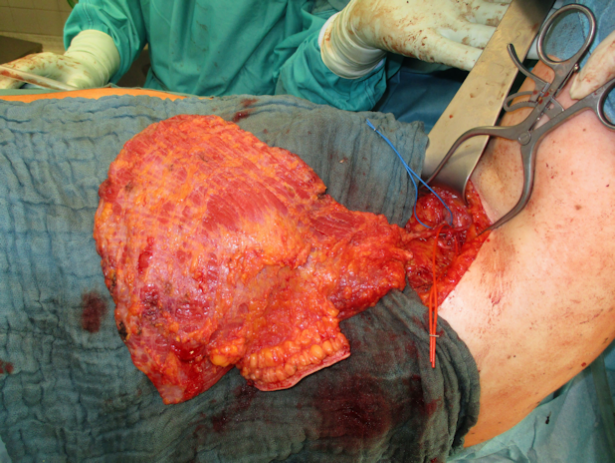
Harvested LD flap

After debridement of the wound base the LD flap was harvested with markings of the venous (blue) and arterial (red) thoracodorsal (TD) vessel (Figure 12 (Fig. 12)). 

**Figure 13:**
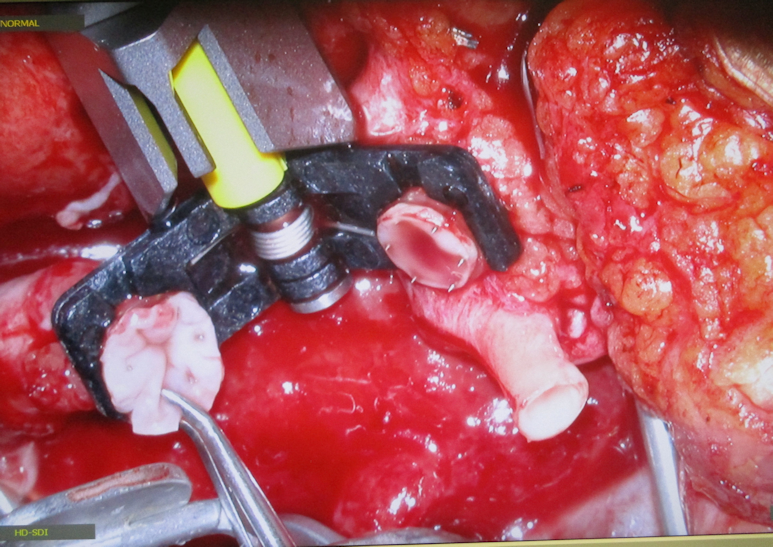
Venous anastomosis perfomed with 3.0 mm coupler

**Figure 14:**
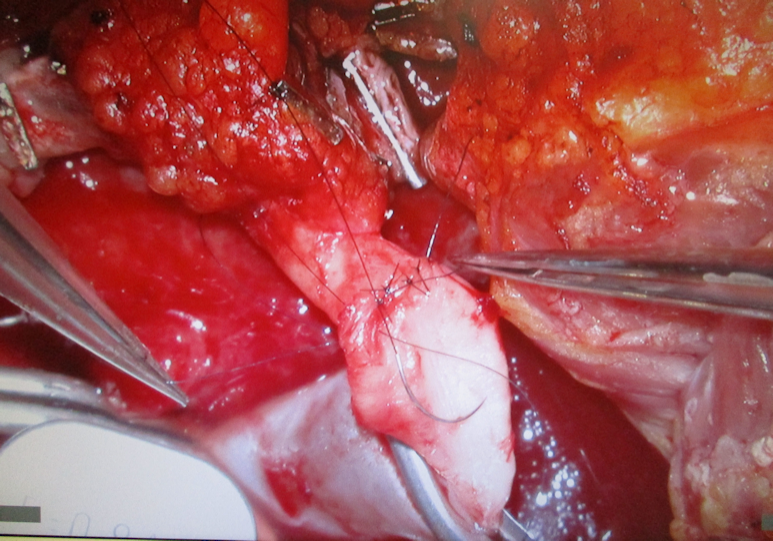
Arterial anastomosis, single button 8.0 Prolene sutures

**Figure 15:**
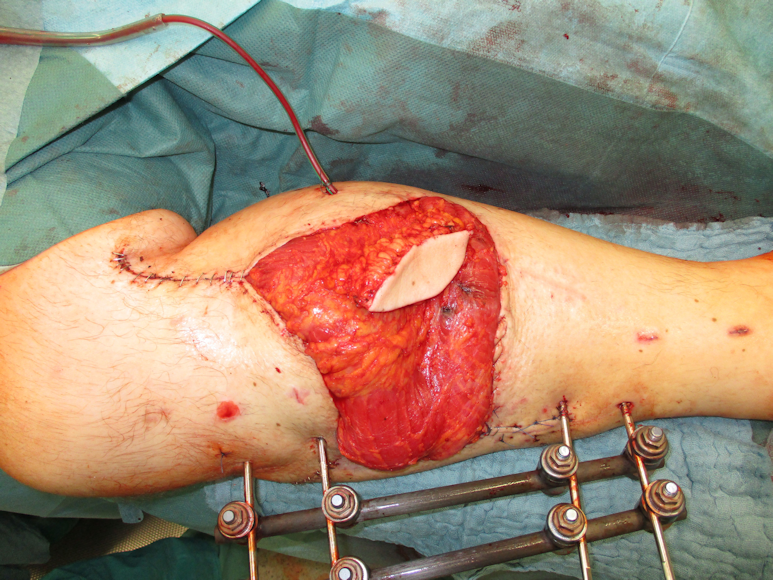
LD flap after anastomosis covering the defect

**Figure 16:**
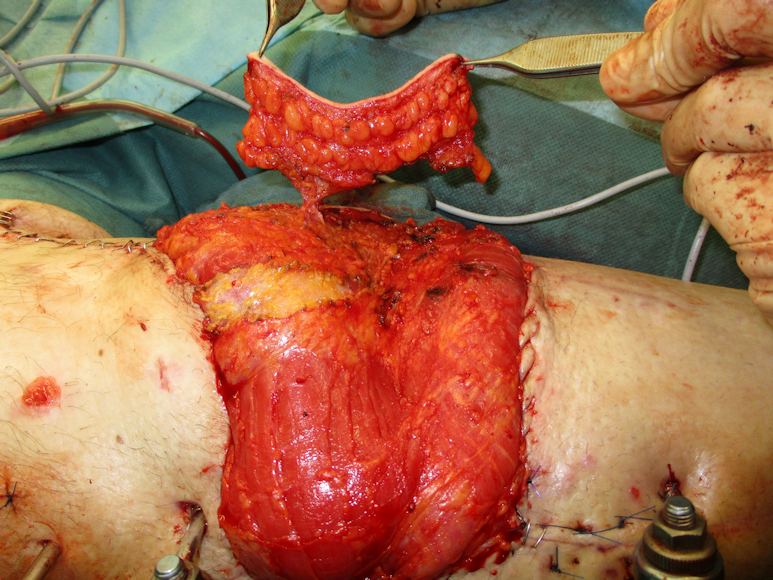
Monitor island with LD flap

Under the surgical microscope, the venous anastomosis was performed with a 3.0 mm venous coupler (Figure 13 (Fig. 13)) whereas the arterial anastomosis was carried out using 8/0 Prolene single-button sutures (Figure 14 (Fig. 14)). After an ischemic time of 56 min the anastomoses were released and an excellent blood flow through the entire latissimus dorsi flap with instant capillarization of the monitor island could be seen (Figure 15 (Fig. 15)). Via magnification and by means of a sterile hand Doppler a perforator was identified on the skin island and the monitor island was prepared leaving only the perforator as a point of attachment (Figure 16 (Fig. 16)). By chosing the borders of the skin island primarily 2 centimeters (cm) behind the anterior border of the LD muscle and 4 cm inferior to the inferior tip of the scapula, preoperative marking of the latissimus dorsi artery perforators (LDAP) is not obligatory and can be conducted intraoperatively after skin island separation as reliable LDAPs derive in this area from the lateral branch of the TD artery. Split skin was transplanted from the left thigh (ratio of 1:1.5) to the latissimus dorsi flap and the monitor island was fixed by two anchoring seams to avoid shearing forces on the perforator. The final examination verified excellent blood circulation of the flap and monitor island (Figure 3 (Fig. 3)). 

## Discussion

Severe crush injuries of the lower extremity due to high force impact require complex soft tissue and bone reconstruction by a well trained interdisciplinary team consisting of skilled orthopedic surgeons and plastic surgeons with profound microsurgery expertise. An early involvement of plastic surgical expertise (emergency room) ensures optimized soft tissue management. The implementation of interdisciplinary reconstructive boards, shared rounds as well as critical complications and case reviewing sessions have shown to improve the treatment outcome and reduce hospitalisation days.

The success of free tissue transfers to cover complex defects depends both on the proper selection of recipient vessels and the excision of the damaged vascular section. Although several promising attempts via drug administration (heparin, botox) have been made to diminish the effects of the endothelial injury, the mechanical impact of the primary trauma is still crucial as changes of all layers of the vessel wall extend well beyond the site of the original injury. Therefore performing the anastomosis from a reliable recipient vessel and outside the zone of injury constitutes a deciding factor for the reconstructive success. In these situations, recipient vessels can be made available for free flap transfer by constructing AV loops from vessels well outside the zone of trauma using long vein grafts, which are divided later for tension free anastomosis to free flap vessels. AV loops produce significantly lesser flap thrombosis than interposition grafts and are excellent choices for complex reconstructions in areas with unsafe and limited local blood supply allowing an anastomosis outside the zone of injury. In context of an interdisciplinary approach, the denovo vessels are to be constructed by the vascular surgery department. As of now, there is no clear consensus about delayed or instant anastomosis after AV loop creation. A two-stage approach allows maturation and observation of the fistula whereas instant anastomosis offers a virgin field and earlier wound coverage. In high risk patients the subsequent procedure is considered to be the safer option as the blood supply provided by the loop is more reliable and the operation time is reduced. Reconsidering the reconstructive algorithm, the two-stage procedure should have been the primary option instead of risking flap loss and the posterior tibial artery by performing an end-to-end anastomosis [7], [8], [9], [11], [13], [14]. 

Concerning the choice of the free flap for reconstruction of lower limb defects there are no established guidelines as of now. A French study compared the outcomes of 47 post-traumatic free flap ALT and LD reconstructions of the lower limb. The LD and ALT group exhibited no significant differences regarding early and late complications and long-term functional outcomes (bone healing, infectious bone complications, flap healing). As for aesthetic outcome and donor-site morbidity, reconstruction using the ALT free flap showed significantly better results [12]. The dictum of preferring muscle flaps when facing chronic infections has been altered as fasciocutaneus flaps demonstrate a similar healing outcome in chronic infections, but facilitate flap elevation for (re)addressing the osteosynthetic material [15]. Compared with muscle flaps (LD, parascapular flap, combined LD and parascapular flaps) ALT flaps showed significant more flap failure in combination with AV loops due to size mismatch of the perforators (mean diameter 2 mm) and the vein graft (mean diameter 5 mm) resulting in higher flow resistance. Therefore axially vascularized flaps with long pedicles allow better adjustment of the lumina and are to be preferred in AV loop reconstruction [16].

Temporary external fixation and NPWT are to be preferred when the surgical team is confronted with unclear extent of soft tissue damage and possible infections (damage control). From a plastic surgeon’s point of view NPWT ensures safe temporary wound sealing to prevent infections. Combined with active wound cleansing, it optimizes soft tissue management and enhances microcirculation and wound preconditioning preceding definitive wound closure [11], [17]. 

The LD flap following the creation of the AV loop showed best surgical and clinical results. Upgraded with a single perforator skin island it presented remarkable visual results as well as a safe Doppler monitoring status without any need to uncover the entire flap and expose the vulnerable healing area [18]. 

Furthermore, when the skin island was no longer needed for observation, it was easily removed during a bedside procedure without requiring any additional surgical treatment or anesthesia.

## Conclusion

Complex osteocutaneus reconstruction of the lower limbs after high force traumata demands a well-established interdisciplinary team trained in finding creative solutions. In order to improve polytrauma treatment plastic reconstructive surgery has to be implemented into the interdisciplinary reconstructive concept of hospitals to provide maximal care.

## Notes

### Competing interests

The authors declare that they have no competing interests.

## Supplementary Material

Contrast CT video
